# Supplementary material for: Metacell-2: a divide-and-conquer metacell algorithm for scalable scRNA-seq analysis
Source: Genome Biol. 2022 Apr 19;23:100. doi: 10.1186/s13059-022-02667-1 (PMC9019975; doi:10.1186/s13059-022-02667-1)
Supplement: Supplementary file 1 — Additional file 1. Supplementary figures and table. [file 13059_2022_2667_MOESM1_ESM.pdf]

Supplementary Figures

Figure S1: More detailed schematics of the MC2 algorithm

The input is a large UMI matrix, MC2 is a recursive two-phase process working as follows: **I**, Preprocessing detecting rare gene modules and metacells based on them and move these directly to the output, **II**, dividing all cells into random piles. **III** – graph partition defines metacells in each pile. **IV**, outlier cells are removed from metacells into specialized piles, used to create additional (rare) metacells (recursively). **V,** MC2 groups metacells into metagroups (recursively). **VI**, metagroups are used as new homogeneous piles. **VII**, Generation of metacells in 2nd iteration piles. **VIII**. Remaining outliers collected from 2nd iteration piles, further grouped into rare metacells, with detection of final outliers. The final output is a set of final metacells and outliers.

Figure S2: MC2 models for PBMC with and without divide and conquer

A. Heatmap showing marker gene expression (log2 normalized compared to median) for the non DAC MC2 model.

B. Heatmap showing marker gene expression (log2 normalized compared to median) for the DAC MC2 model.

C. Gene expression per metacell for select T-cell genes as in Baran et al.

Figure S3: Bone marrow MC models

A. Heatmap showing marker gene expression (log2 normalized compared to median) for the bone marrow global model.

B. Marker gene expression for the MC2 models constructed using only HSC/MPP cells.

Figure S4: Organogenesis cell types vs. metacell clusters

Matrix is showing the number of cells in each combination of metacell cluster and organogenesis atlas cell type.

Figure S5: Metacell structure within broad cell types

Marker heat map (log2 gene expression normalized to the median over all metacell) is shown for metacells within the epithelial (29-31, A) and endothelial (18-19, B) metacell clusters. Rich combinatorial and quantitative variation is observed within each of the broader cell types, setting the stage for in-depth follow up analysis.

Table T1: Default main parameters

Default values for the main parameters controlling the Metacell2 pipeline.

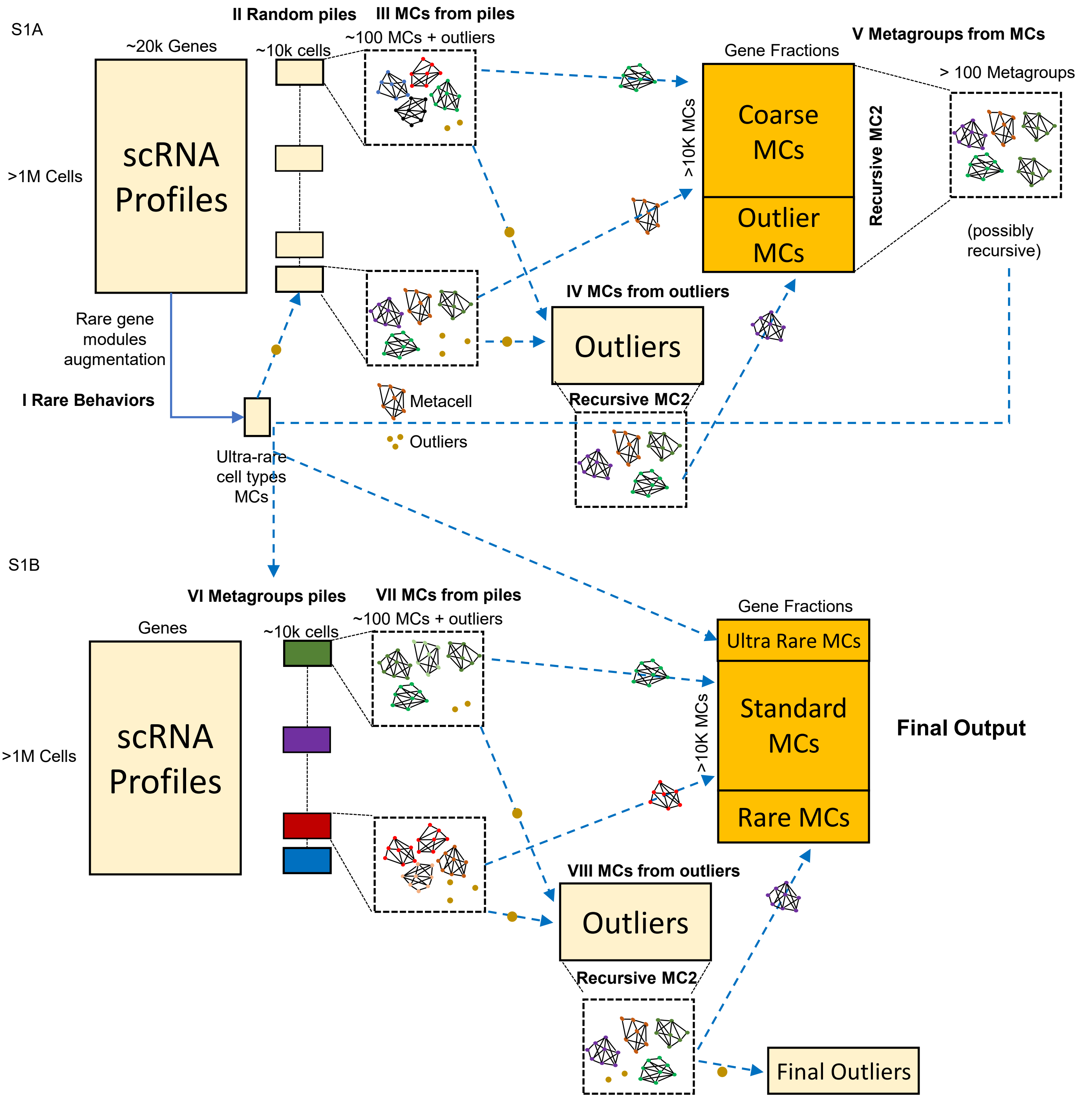

Ben Kiki Fig S1

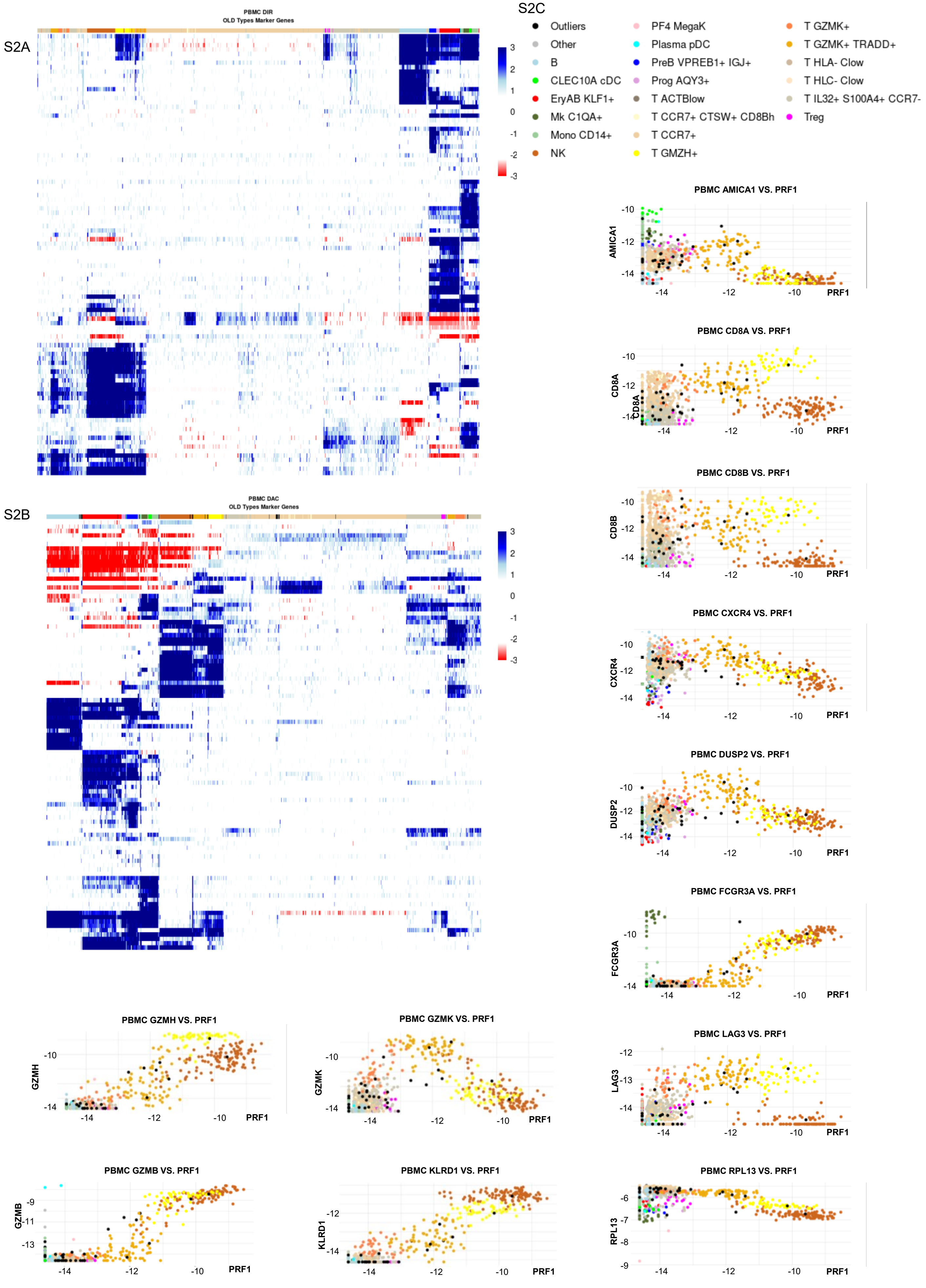

Ben Kiki Fig S2

S3A

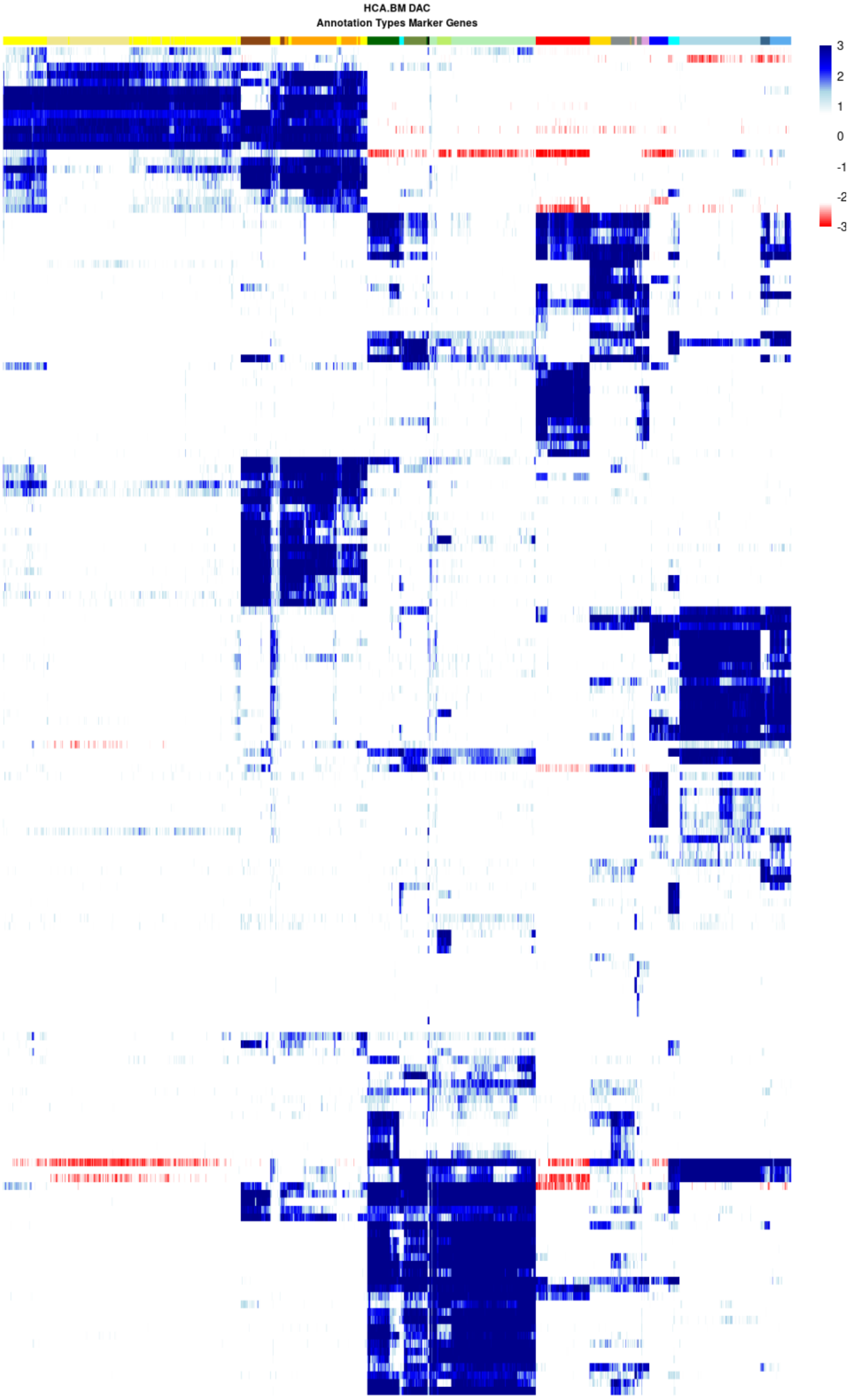

S3B

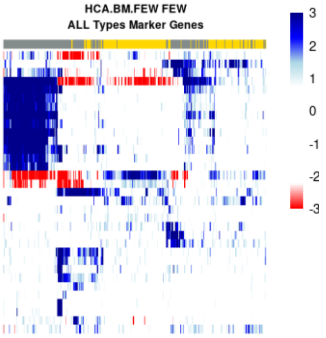

Ben Kiki Fig S3

S4

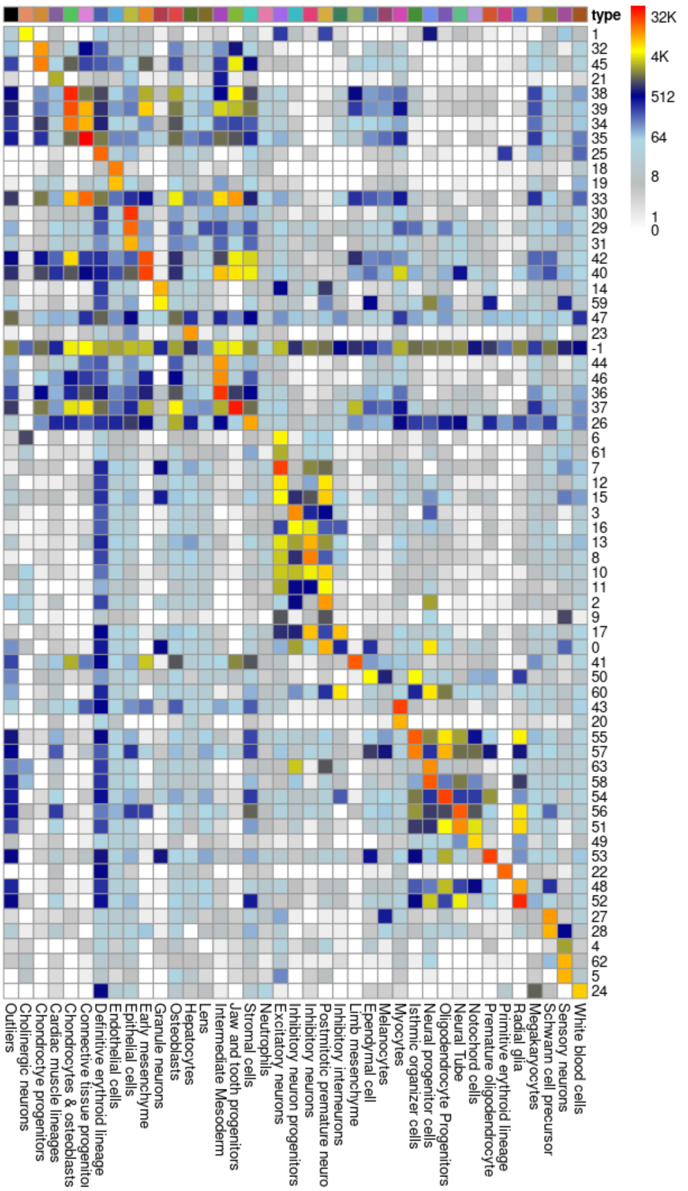

Ben Kiki Fig S4

S5A

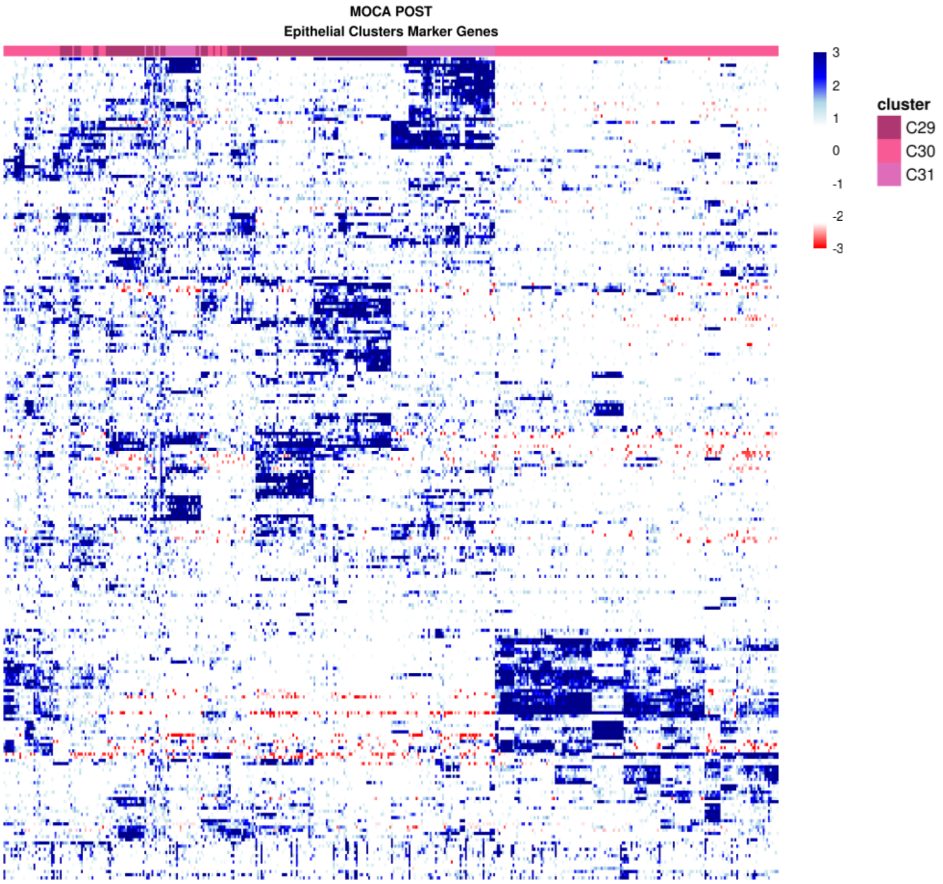

S5B

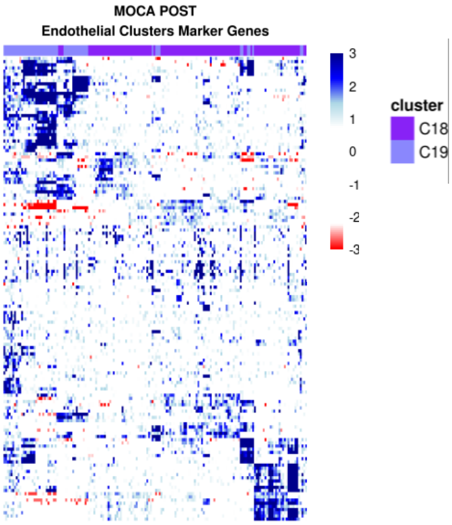

Ben Kiki Fig S5

Metacells Main Adjustable Parameters

Parameter	Default	Description
rare_max_gene_cell_fraction	0.001 (0.1%)	The maximal fraction of the cells where a gene is expressed to be considered "rare"
rare_min_gene_maximum	7	The minimal maximum-across-all-cells value of a gene to be considered as a candidate for rare gene modules
rare_min_genes_of_modules	4	The minimal number of genes in a rare gene module
rare_min_cells_of_modules	12	The minimal number of cells in a rare gene module
rare_min_cell_module_total	4	The minimal number of UMIs of a rare gene module in a cell to be considered as expressing the rare behavior
rare_max_cells_of_random_pile	48	The maximal mean number of cells in a random pile for a rare gene module to be considered rare
feature_downsample_min_samples	750	The minimal samples to use for downsampling the cells for computing "feature" genes
feature_downsample_min_cell_quantile	0.05 (5%)	The minimal quantile of the cells total size to use for downsampling the cells for computing "feature" genes
feature_downsample_max_cell_quantile	0.5 (50%)	The maximal quantile of the cells total size to use for downsampling the cells for computing "feature" genes
feature_min_gene_total	50	The minimal number of downsampled UMIs of a gene to be considered a "feature"
feature_min_gene_top3	4	The minimal number of the top-3rd downsampled UMIs of a gene to be considered a "feature"
feature_min_gene_relative_variance	0.1	The minimal relative variance of a gene to be considered a "feature"
forbidden_gene_names	None	Genes forbidden from being a “feature”
forbidden_gene_patterns	None	Genes forbidden from being a “feature”
target_metacells_in_pile	100	The target number of metacells in a pile, allowing us to directly compute it
min_target_pile_size	10000	Minimal target pile size (in cells), even if resulting with more metacells per pile
max_target_pile_size	30000	Maximal target pile size (in cells), even if resulting with less metacells per pile
max_cell_size	None	The maximal cell size (total UMIs) to use
max_cell_size_factor	X 2	The maximal cell size as a factor of the median cell size
cell_sizes	__x__|sum (Sum of UMIs)	The size of each cell for computing each metacell's size
target_metacell_size	160000	The target total metacell size (in UMIs)
knn_k	None (Automatic)	The target K for building the K-Nearest-Neighbors graph
min_knn_k	30	The minimal target K for building the K-Nearest-Neighbors graph
candidates_cooldown_pass	0.02	By how much (as a fraction) to cooldown the temperature after doing a pass on all the nodes
candidates_cooldown_node	0.25	By how much (as a fraction) to cooldown the node temperature after improving it
candidates_cooldown_phase	0.75	By how much (as a fraction) to reduce the cooldown each time we re-optimize a slightly modified partition
candidates_min_metacell_cells	12	The minimal number of cells in a metacell, below which we would merge it
must_complete_cover	FALSE	Whether to force 100% coverage (disable outliers detection)
deviants_min_gene_fold_factor	3 (X 8)	The minimal fold factor for a gene to indicate a cell is "deviant"
deviants_max_gene_fraction	0.03 (3%)	The maximal fraction of genes to use to indicate cell are "deviants"
deviants_max_cell_fraction	0.25 (25%)	The maximal fraction of cells to mark as "deviants"
dissolve_min_metacell_cells	12	The minimal number of cells in a metacell, below which we would dissolve it
random_seed	None (Irreproducible)	Optional integer random seed for reproducible results
