# Supplementary material for: Metacell-2: a divide-and-conquer metacell algorithm for scalable scRNA-seq analysis
Source: Genome Biol. 2022 Apr 19;23:100. doi: 10.1186/s13059-022-02667-1 (PMC9019975; doi:10.1186/s13059-022-02667-1)
Supplement: Supplementary file 2 — Additional file 2. Review history. [file 13059_2022_2667_MOESM2_ESM.docx]

Review History

**First round of review**

**Reviewer 1**

**Were you able to assess all statistics in the manuscript, including the appropriateness of statistical tests used?**

Yes: No issues

**Were you able to directly test the methods?**

Yes

**Comments to author:**

Ben-Kiki et al. describe Metacell 2, an updated version of the Metacell algorithm from the same group.

The Metacell algorithm is a unique and refreshing take on single-cell analysis. It is based on the idea that, in datasets with very large numbers of cells, it is likely that essentially the same cell states reoccur in close neighboprhoods on the manifold. If those near-identical cell states can be identified and pooled together to form metacells, then the low sensitivity (sparsity) of the raw data can be mitigated. Satisfyingly, the sum of N multinomial distributions having different total counts n1, n2, … nn (e.g. different sampling depths per cell) but the same probability vector p1, p2, … pn (by the assumption that the cells are near-identical) is still multinomial with n = sum(n1, n2, … nn). Hence, the statistical properties of metacells remain unchanged and it's as if we just sampled a single much larger cell without the issues of noisy data and many zeros. In particular, this approach permits detection of low-expressed genes in single metacells, which would go undetected in single cells.

Metacell is not intended as a complete single-cell analysis pipeline, but rather as a preprocessing step that is similar to (but more principled than) imputation algorithms.

Metacell 2 is far more scalable than version 1, and is implemented in Python rather than R. It comes with extensive documentation and several vignettes (tutorials) to introduce new users. Installation was easy and I had no trouble getting started.

Overall, Metacell 2 is an important addition to the toolbox of single-cell analysis and I have no hesitation recommending publication. I do have a couple of questions:

1. The feature selection step is crucial, at least for standard PCA-based pipelines. It was unclear from the methods how many features were used, how they were selected, and if they were re-selected during divide and conquer. I find from my own analysis that a divide-and-conquer version of the standard PCA-KNN-Louvain pipeline can work very well provided that new features are selected in each round. This allows very different cell types to be analyzed each in their own optimal subspaces.

2. What distance metric was used in graph construction, and why? What were the edge weights?

3. How does the algorithm compare with Poisson pooling? (https://www.biorxiv.org/content/10.1101/217737v3). Both approaches are based on local pooling of data, but Metacell contracts the dataset to coarser metacells, whereas Poisson pooling retains the full dataset with pooling among KNN neighbors. What are the implications of these differences, and differences in implementation?

**Reviewer 2**

**Were you able to assess all statistics in the manuscript, including the appropriateness of statistical tests used?**

Yes: The statistical approach is sound. I have specific questions in the comments to authors.

**Were you able to directly test the methods?**

No

**Comments to author:**

This paper describes metacell2, a computational tool and extension of the previously published metacell tool, which aggregates read information across transcriptionally proximal cells. The estimation of metacells is an established first-line analysis strategy for reducing the complexity and size of single-ell variation datasets. Further extensions and refinements of this approach are of interest to the community. The proposed advances are mainly technical, although the improved computational complexity will be of interest to users. I also feel that the demonstration of the added value and the benchmarking of metacell2 should be improved. The paper could would also benefit from careful editing to improve clarity. Several figures are also rendered poorly.

Major comments

* The presentation of the differences between MC1 and MC2 is currently focused on the scaling behavior and robustness. Based on the presentation, I struggle to understand which additional aspects are uniquely enabled by MC2 versus which aspects of the results could already be achieved with the predecessor (bar the difference in computational complexity). It would be helpful to discuss these differences more carefully, e.g. rare cell type identification and outlier detection, etc. These features seem to be offered by MC1 as well.

* The analysis of the computational complexity (Fig 3a) considers the comparison to a PCA + Leiden workflow. This case could be stronger and an explicitly comparison to MC1 would be beneficial. I also wonder whether the analyses of the robustness of Mc2 (Fig. 2) should consider varying the number of piles in the divide & conquer scheme. Do larger numbers of piles result in reduced accuracy as sets of cells are treated independently ?

* The steps taken to improve the computational scalability should be defined more clearly. First, "arbitrary scaling" seems a somewhat optimistic description for an algorithm with O(Nlog(N)) scaling. I also miss information on whether and how the analyses within individual piles is parallelized in the software implementation of MC2.

* Data representation and downstream analysis. The utility of MC2 will depend on the infrastructure and embedding to use these representations for downstream analyses. This aspect would deserver more attention in the paper.

Minor comments

* Unclear package dependencies. The abstract indicates the method is implemented within scanpy. The introduction then points to interoperability with multiple frameworks.

* It is stated that the piling approach is parallelizable. Is parallelization offered by the software ?

**Authors Response**

**Point-by-point responses to the reviewers’ comments:**

Reviewer #1:

Ben-Kiki et al. describe Metacell 2, an updated version of the Metacell algorithm from the same group.

The Metacell algorithm is a unique and refreshing take on single-cell analysis. It is based on the idea that, in datasets with very large numbers of cells, it is likely that essentially the same cell states reoccur in close neighboprhoods on the manifold. If those near-identical cell states can be identified and pooled together to form metacells, then the low sensitivity (sparsity) of the raw data can be mitigated. Satisfyingly, the sum of N multinomial distributions having different total counts n1, n2, ... nn (e.g. different sampling depths per cell) but the same probability vector p1, p2, ... pn (by the assumption that the cells are near-identical) is still multinomial with n = sum(n1, n2, ... nn). Hence, the statistical properties of metacells remain unchanged and it's as if we just sampled a single much larger cell without the issues of noisy data and many zeros. In particular, this approach permits detection of low-expressed genes in single metacells, which would go undetected in single cells.

Metacell is not intended as a complete single-cell analysis pipeline, but rather as a preprocessing step that is similar to (but more principled than) imputation algorithms.

Metacell 2 is far more scalable than version 1, and is implemented in Python rather than R. It comes with extensive documentation and several vignettes (tutorials) to introduce new users. Installation was easy and I had no trouble getting started.

Overall, Metacell 2 is an important addition to the toolbox of single-cell analysis and I have no hesitation recommending publication. I do have a couple of questions:

*We thank the reviewer for their support and for taking the time to provide the comments below.*

1. The feature selection step is crucial, at least for standard PCA-based pipelines. It was unclear from the methods how many features were used, how they were selected, and if they were re-selected during divide and conquer. I find from my own analysis that a divide-and-conquer version of the standard PCA-KNN-Louvain pipeline can work very well provided that new features are selected in each round. This allows very different cell types to be analyzed each in their own optimal subspaces.

*We thank the reviewer for highlighting this important point. We added a section to the methods describing feature selection as the original methods was indeed very implicit in that respect. In short: feature selection is performed independently within each pile, and this is indeed critical for the sensitivity of the algorithm. To facilitate automated feature selection within piles we use a strategy very similar to the original single-pile metacell algorithm, but allow the user to provide a global list of genes that should be omitted for the feature list (since from our experience, the main issue is with excluding batch-related genes, or genes linked with biological behavior that we wish to normalize such as the cell cycle).*

2.	What distance metric was used in graph construction, and why? What were the edge weights?

*Similarly to the previous point, the original version lacked details as we rely on a similar construction to the original metacell algorithm. This is now clarified in the methods section. Thank you!*

3.	How does the algorithm compare with Poisson pooling?

*(https://www.biorxiv.org/content/10.1101/217737v3). Both approaches are based on local pooling of data, but Metacell contracts the dataset to coarser metacells, whereas Poisson pooling retains the full dataset with pooling among KNN neighbors. What are the implications of these differences, and differences in implementation?*

*This is an important comment that highlights the differences between smoothing and imputation strategies for scRNA-seq and the micro-clustering strategy of metacells. We have added some comments regarding these differences to the discussion and note that some comparisons of K-nn and diffusion-based imputation were already included in the Baran et al manuscript. Also note that as datasets become very large, single cell imputation strategies are becoming practically non-feasible.*

Reviewer #2: This paper describes metacell2, a computational tool and extension of the previously published metacell tool, which aggregates read information across transcriptionally proximal cells. The estimation of metacells is an established first-line analysis strategy for reducing the complexity and size of single-ell variation datasets. Further extensions and refinements of this approach are of interest to the community. The proposed advances are mainly technical, although the improved computational complexity will be of interest to users. I also feel that the demonstration of the added value and the benchmarking of metacell2 should be improved. The paper could would also benefit from careful editing to improve clarity. Several figures are also rendered poorly.

*We thank the reviewer for their support and for the constructive and useful comments below. We revised the manuscript with some additional details and analysis as suggested.*

Major comments

*	The presentation of the differences between MC1 and MC2 is currently focused on the scaling behavior and robustness. Based on the presentation, I struggle to understand which additional aspects are uniquely enabled by MC2 versus which aspects of the results could already be achieved with the predecessor (bar the difference in computational complexity). It would be helpful to discuss these differences more carefully, e.g. rare cell type identification and outlier detection, etc. These features seem to be offered by MC1 as well.

*We thank the reviewer for highlighting this point. We have improved the relevant discussion section to clarify the relationships between MC1 and MC2, not only regarding the scalability (which is indeed a key point!), but also with respect to major improvement with outlier filtering and graph partitioning. With respect to rare gene detection, this is not a real issue for small datasets such as those accessible via MC1 – the crux with MC2 rare cell type detection is that its sensitivity is maintained while scalability is ensured.*

*	The analysis of the computational complexity (Fig 3a) considers the comparison to a PCA + Leiden workflow. This case could be stronger and an explicitly comparison to MC1 would be beneficial. I also wonder whether the analyses of the robustness of Mc2 (Fig. 2) should consider varying the number of piles in the divide & conquer scheme. Do larger numbers of piles result in reduced accuracy as sets of cells are treated independently?

*This is an important point, and we have followed up accordingly. We have tested systematically the impact of changing pile sizes on runtime and on accuracy (Fig p2p_1, also now Fig 3C). We found that indeed, pile size can impact accuracy when the total number of target metacells within a pile (which is also dependent on the number of UMIs per cell!) goes below a certain threshold. This is something the algorithm should generally avoid, and our original default parameter of 10,000 cells per pile may be suboptimal for datasets that have very low sequencing/UMI depth. Following the reviewer suggestion, we therefore improved MC2 pile size determination. The algorithm selects a pile size that guarantees (given the target number of UMIs per MC), that the expected number of MCs per pile will be (by default) 100. Pile sizes are still bounded between 10,000 and 30,000. Note that for typical 10X datasets and default MC size, the pile size will still be 10,000, but for experiments profiling very small cells, the more careful adaptation of pile size can have a positive impact on accuracy (at the cost of increased runtime!). For the organogenesis atlas, we have chosen to use a target of 50.*

*Fig_p2p_1: showing the effect of using piles of increasing size on runtime and metacells’ inner variance.*

*	The steps taken to improve the computational scalability should be defined more clearly. First, "arbitrary scaling" seems a somewhat optimistic description for an algorithm with O(Nlog(N)) scaling. I also miss information on whether and how the analyses within individual piles is parallelized in the software implementation of MC2.

*Point well taken. We changed the terminology regarding scaling: the algorithm scales in an unlimited fashion with respect to memory, but it is nlogn in terms of total cpu time. Regarding parallelization – we clarified that we are not parallelizing inside piles (with the exception of implicit matrix operations).*

*	Data representation and downstream analysis. The utility of MC2 will depend on the infrastructure and embedding to use these representations for downstream analyses. This aspect would deserve more attention in the paper.

*We thank the reviewer for this point. We added a method section discussion downstream processing of MC2 output. Additional information is available in the published python package documentation.*

Minor comments

*	Unclear package dependencies. The abstract indicates the method is implemented within scanpy. The introduction then points to interoperability with multiple frameworks.

*In the new methods section discussing downstream processing suggest how MC2 output can be further processed in general. This can be done using e.g., scanpy, Seurat or other tools. The integration into scanpy mostly relates to native processing of scanpy annotated data representation – but other formats are easily imported. For generality (and to meet length restriction in the abstract) – we omitted the direct reference to scanpy from the abstract.*

*	It is stated that the piling approach is parallelizable. Is parallelization offered by the software ?

*Indeed. MC2 is running parallel processes to analyze piles – the number of processes depends on the machine and user defined parameters.*

**Second round of review**

**Reviewer 1**

The authors have responded satisfactorily to all my questions and those of the other reviewer. I have no further objections, and wish to thank the authors for their work and for providing such an important tool to the community.
